# Analysis of genomic copy number variations through whole-genome scan in Chinese Qaidam cattle

**DOI:** 10.3389/fvets.2023.1148070

**Published:** 2023-03-31

**Authors:** Yangkai Liu, Yanan Mu, Wenxiang Wang, Zulfiqar Ahmed, Xudong Wei, Chuzhao Lei, Zhijie Ma

**Affiliations:** ^1^Plateau Livestock Genetic Resources Protection and Innovative Utilization Key Laboratory of Qinghai Province, Academy of Animal Science and Veterinary Medicine, Qinghai University, Xining, China; ^2^Key Laboratory of Animal Genetics and Breeding on Tibet Plateau, Ministry of Agriculture and Rural Affairs, Xining, China; ^3^Key Laboratory of Animal Genetics, Breeding and Reproduction of Shaanxi Province, College of Animal Science and Technology, Northwest A&F University, Xianyang, China; ^4^Faculty of Veterinary and Animal Sciences, University of Poonch Rawalakot, Rawalakot, Pakistan

**Keywords:** Qaidam cattle, whole genome resequencing, copy number variation (CNV), genome selection, population structure

## Abstract

Qaidam cattle (CDM) are indigenous breed inhabiting Northwest China. In the present study, we newly sequenced 20 Qaidam cattle to investigate the copy number variants (CNVs) based on the ARS-UMD1.2 reference genome. We generated the CNV region (CNVR) datasets to explore the genomic CNV diversity and population stratification. The other four cattle breeds (Xizang cattle, XZ; Kazakh cattle, HSK; Mongolian cattle, MG; and Yanbian cattle, YB) from the regions of North China embracing 43 genomic sequences were collected and are distinguished from each of the other diverse populations by deletions and duplications. We also observed that the number of duplications was significantly more than deletions in the genome, which may be less harmful to gene formation and function. At the same time, only 1.15% of CNVRs overlapped with the exon region. Population differential CNVRs and functional annotations between the Qaidam cattle population and other cattle breeds revealed the functional genes related to immunity (*MUC6*), growth (*ADAMTSL3*), and adaptability (*EBF2*). Our analysis has provided numerous genomic characteristics of some Chinese cattle breeds, which are valuable as customized biological molecular markers in cattle breeding and production.

## 1. Introduction

Domestic cattle are one of the important animals that have been used as a source of materials for production and development by human civilization. Approximately 850,000 years ago, domestic cattle diverged into two groups, namely, humpless taurine (*Bos Taurus*) and humped indicine (*Bos Indicus*) (1, 2). Moreover, environmental factors, geographical isolation, and human activities also contributed to the development of present-day cattle. Through a long period of domestication, megabases (Mb) of DNA gradually enriched the genomic diversity among cattle breeds (3).

As of 2021 (4), there are already 55 Chinese indigenous breeds. The Qaidam cattle (CDM) is one of the 55 breeds reared in Northwest China (36°21'−39°23' N, 90°30'−99°30' E, Qinghai Province, China), where the drought (annual precipitation <200 mm) and high altitude (2,600–3,000 m) environment is predominant, and these conditions made the Qaidam cattle breed have more stress resistance, rough feeding tolerance, and environmental adaptability. During the Yuan dynasty (AD 1271–1638) period, the Mongolian army introduced the Mongolian cattle (*Bostaurus*) into the present-day Qinghai and Gansu Provinces of China during a southward invasion, which might have influenced the breeding herds of the present Qaidam cattle. Paternal and maternal diversity studies indicated that Qaidam cattle included two lineages (1, 5). The autosomal genetic evidence suggests that the Qaidam cattle was closer to Mongolian cattle, which is a hybrid of *Bos Taurus* × *Bos Indicus* (1). The purebred Qaidam cattle have not been effectively protected for their low economic returns. There was a 47.80% decrease in the Qaidam cattle population by 2006 compared to the 1981 Qaidam cattle population (6).

The copy number variations (CNVs) are defined as the deletion or duplication of a genome copy number, ranging from 50 bp to several Mb in length (7). As compared to SNP mutations, the CNV fragments are large in length and cover a wider range of genomes that have broader prospects in studying animal genetics and breeding application. Recently, next-generation genome sequencing technologies have been continuously used to detect the genome-wide CNVs of livestock (8, 9). However, numerous genomic studies exploring CNVs in commercial cattle breeds have underestimated the role of native breeds in the adaptation process (10, 11).

In the present study, we performed a genome-wide CNV analysis using genomic resequencing data in six Chinese cattle breeds. The purpose was to generate a comprehensive CNV landscape in Qaidam cattle to investigate and compare the diversity and population–genetic properties of the CNV regions (CNVRs) among them and to explore the diverse selection patterns involved with the CNV genes for local adaptation in Chinese native cattle.

## 2. Materials and methods

### 2.1. Genome resequencing and samples collection

Qaidam Basin is the highest basin in China with an altitude of 2,600–3,000 m and is located in the northwest region of the Qinghai Province and the northeast region of the Qinghai–Tibet Plateau. The climate of the basin is characterized as extremely dry and cold, with an annual average precipitation of <200 mm and an annual average temperature of ~3.0–6.5°C. To reflect the sample representativeness of the Qaidam cattle, 20 samples were collected from five different counties/cities (Dulan, Golmud, Mangya, Wulan, and Dachaidan) in the Qaidam Basin (Supplementary Table 1, **Figure 2A**).

The ear tissues of selected samples were used for DNA extraction by the standard phenol–chloroform protocol. Genomic DNA was constructed into 350-bp libraries and sequenced using Illumina NovaSeq at Novogene Bioinformatics Institute (Beijing, China). Moreover, 42 publicly available data of four Chinese cattle breeds were downloaded in this study (10 Mongolian cattle, MG; 9 Xizang cattle, XZ; 15 Yanbian cattle, YB; and 8 Kazakh cattle, HSK) (Supplementary Table 2). It is worth noticing that the resequencing data of one Xizang cattle (Sample ID: Xizang9) was offered by the Key Laboratory of Animal Genetics, Breeding and Reproduction of Northwest A&F University (Supplementary Table 2).

### 2.2. Genome data generation and CNV calling

Read pairs were aligned to the *B. taurus* reference assembly (ARS-UCD1.2) using the Burrows–Wheeler Aligner (BWA) program with default parameters (12). Then, CNVcaller (13) was applied to call the CNV in each individual. First, to create a *B. taurus* reference database, the ARS-UCD1.2 was split and the overlapping windows were recommended as 800 bp (13). Second, the reads number in each window was calculated, and high similarity (≧97%) reads were merged into segments of the autosomes. Third, the GC bias was used to standardize the copy number in each window, and it was used to classify the different genotypes of each sample. Finally, various steps of CNVcaller filtering parameters were carried out: -f 0.1 -h 3 -r 0.1; a Silhouette score of > 0.6; the length of CNVR of ≤ 50 kb (deletion and both), with the length of CNVR of <500 kb (duplication) (15).

### 2.3. Breed/population differentiation

Principal component analysis (PCA) was used to stratify and cluster the close breeds/populations, which plays a positive role in understanding the genetic differences among cattle subpopulations. According to the smartPCA module of EIGENSOFT (Program 2006), the PCA calculation was performed based on the four different CNVR datasets.

### 2.4. Differential CNVR identification

We calculated *V*_ST_ (15) between Qaidam cattle and the other four cattle breeds (XZ, YB, HSK, and MG) to identify the differential CNVRs. The *V*_ST_ is a method to calculate selection between populations similar to the *F*_ST_ method. The formula is *V*_ST_ = (*V*_T_ – *V*_S_)/*V*_T_, where *V*_T_ represents the variance among all the unrelated individuals and *V*_S_ is the average variance within each population, weighted for population size (16). Finally, the top 1% gene cluster of the *V*_ST_ method was kept out by the outlier method.

The ANNOVAR was applied to annotate the CNVRs in our results (14). Further, the Kyoto Encyclopedia of Genes and Genomes (KEGG) and gene ontology (GO) analysis were performed on the candidate CNV genes by KOBAS 3.0. Since the enriched terms were retained with a *p*-value of <0.05, we preferred showing some of the top pathways; for more information, please see Supplementary Tables.

## 3. Results

### 3.1. CNV discovery and CNVR set statistics

We collected 63 Chinese cattle whole genomes representing five breeds from the north and northwest regions of China, including 20 Qaidam cattle, 10 Mongolian cattle, 15 Yanbian cattle, nine Kazakh cattle, and nine Xizang cattle (**Figure 2A**, Supplementary Table 2). The mean sequencing depth was performed to 12-fold coverage of the *Bos taurus* genome (Supplementary Table 2). Among the 63 genomes, we newly sequenced 20 Qaidam samples and one Xizang sample at ~9-fold coverage each (Supplementary Table 2), and the other 42 genomic sequences were available online.

**Figure 1:**
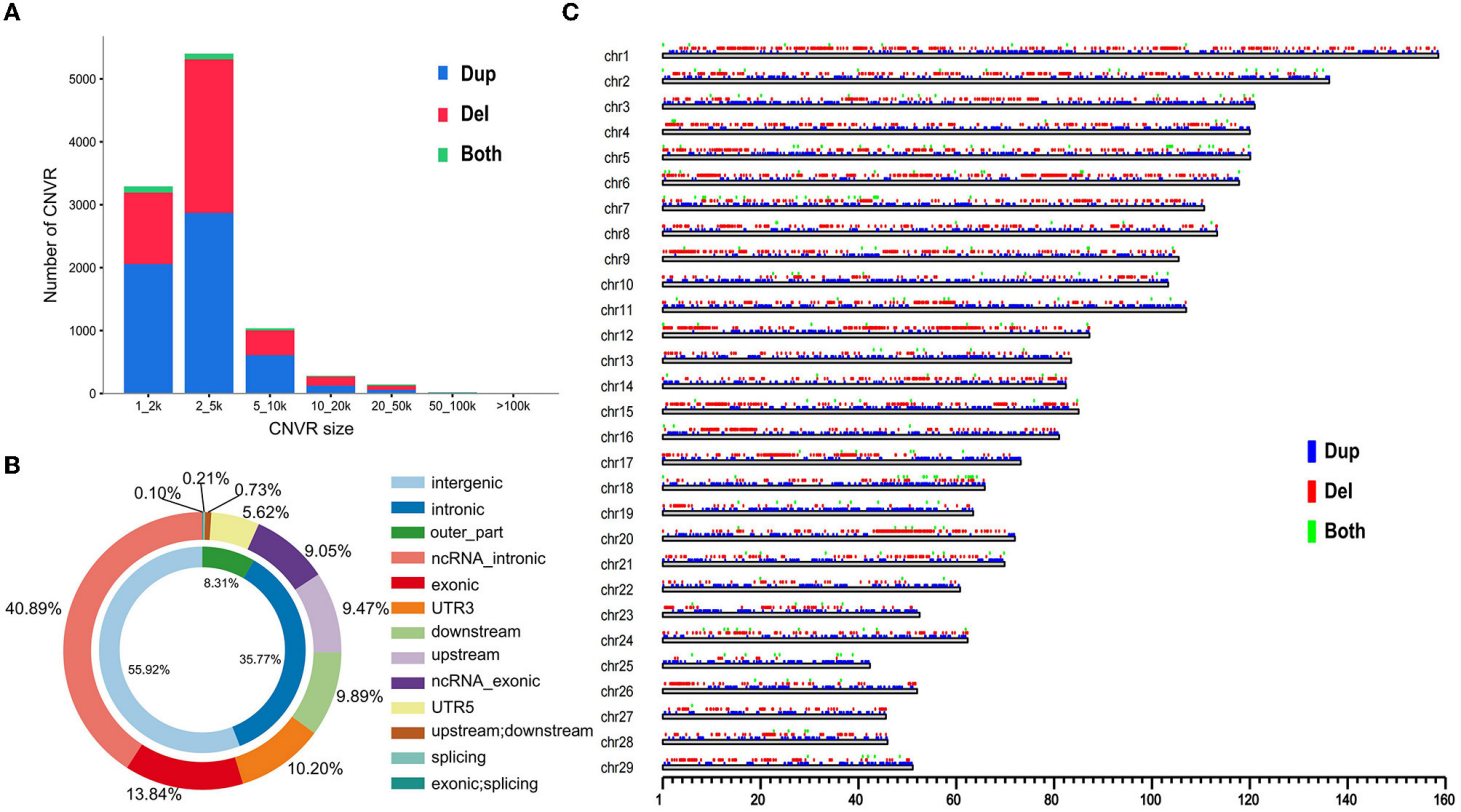
Genomic diversity and distribution of CNVRs. **(A)** The number of the detected CNVR. **(B)** Annotation of CNVRs with various genomic features. The inner circle indicates the intronic region, intergenic region, and the remaining set of functional regions (outer_part). The outer circle includes ncRNA intronic; exonic; UTR3; downstream; upstream; ncRNA exonic; UTR6; splicing; upstream and downstream; exonic and splicing. **(C)** The autosomal distribution of CNVRs. The location with different colors represent duplication (blue), deletion (red), and both duplication and deletion (green).

We applied a read–depth-based bio-software (CNVcaller) to discover autosomal CNVs among individuals relative to the ARS-UCD1.2 reference genome. We generated the CNVR datasets from each cattle breed. The CNVR set contained 10,178 CNVRs, which were detected from 63 cattle genome datasets. There were 5,743 duplication CNVRs; 4,187 deletion CNVRs; and 248 both duplication and deletion CNVRs (Supplementary Table 3). Here, 10,178 CNVRs (duplication, deletion, and both duplication and deletion) were divided into different length groups (Figure 1A). The CNVRs annotation showed that the number of CNVRs was 5,398 (53.04%), which were detected in 2–5 kb size. It was observed that 55.92% CNVRs were located in the intergenic region followed by the intron region (35.77%). However, only 1.16 % CNVRs were detected in the coding exonic region (Figure 1B). And the CNVRs distribute randomly in the chromosome both in number and length (Figure 1C).

### 3.2. Population structure

**Figure 2:**
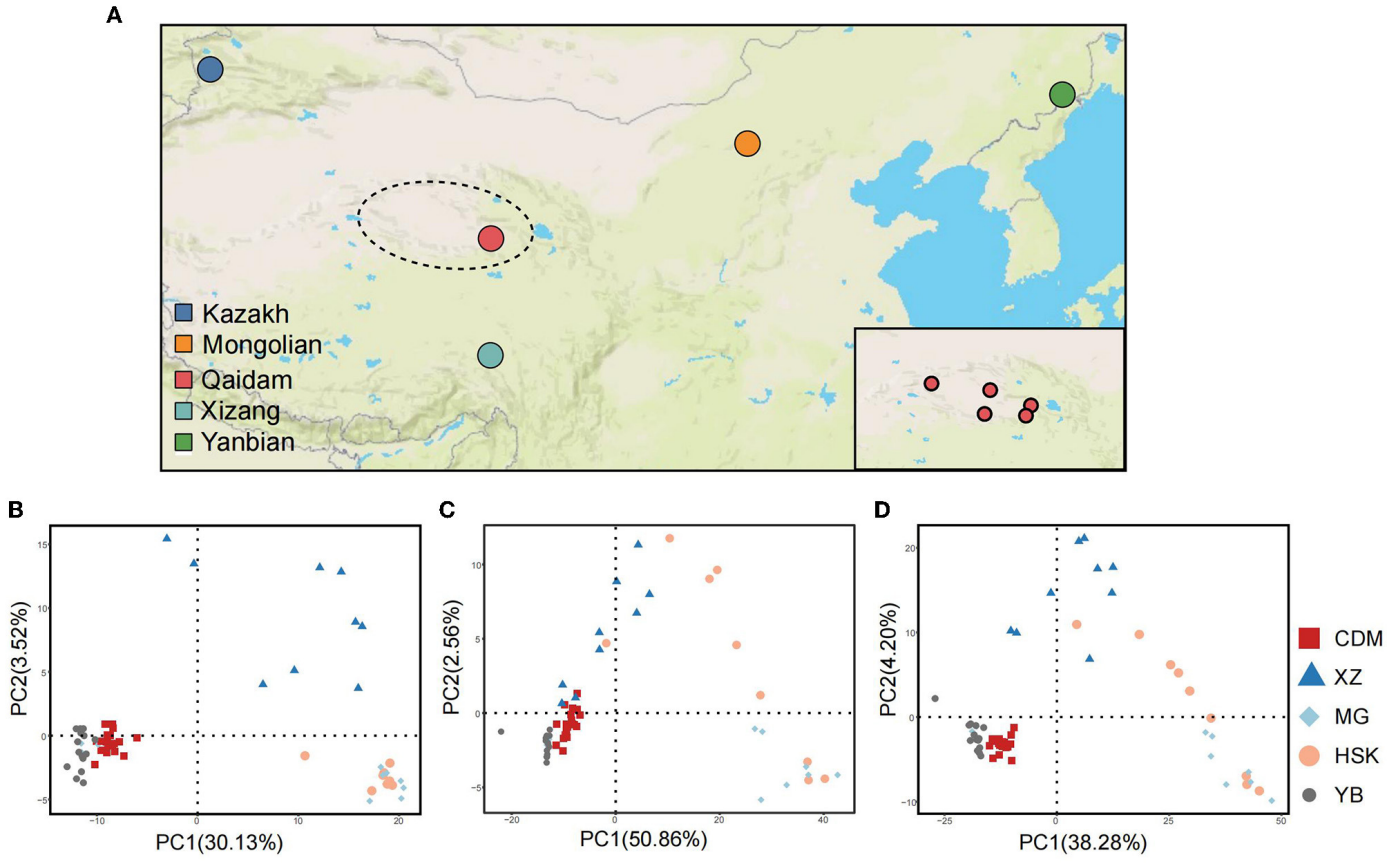
Geographic distribution and population stratification in five Chinese cattle. **(A)** The major geographical locations of the five Chinese cattle breeds. The dotted circle represents the Qaidam Basin, which is the main geographical distribution area of Qaidam cattle. In this study, Qaidam cattle were selected from five geographical locations along the Qaidam Basin, as shown in the lower right corner, which are Dulan, Golmud, Mangya, Wulan, and Dachaidan. **(B)** The principal component analysis (PCA) was derived from CNVRs (deletions). **(C)** PCA derived from CNVRs (duplications). **(D)** PCA derived from all CNVRs in five population genomes.

With the effect of balancing selection, abundant polymorphisms of the genomic copy number variation are found in Chinese *Bos taurus*. A principal component analysis (PCA) was carried out with an obvious distinction from deletions (Figure 2B), duplications (Figure 2C), and total CNVRs datasets (Figure 2D). Qaidam cattle population is broadly distinguished from Mongolian, Kazakh, and Xizang breeds and closely clustered with the Yanbian breed. The PC1 explained ~30.13–50.86% of the genetic variation. For deletions, PC1 (30.13% of the variance) could separate Qaidam and Yanbian breeds from the other breeds, and PC2 (3.52% of the variance) could distinguish Xizang cattle (Figure 2B) from the other breeds (Kazakh and Mongolian cattle). Compared to deletions, duplications separated Qaidam cattle from all other breeds, in general, as shown in the PCA, but its clustering had less accuracy (Figure 2C). The effect of the PCA using both CNVR types was not optimistic in the clustering populations (Supplementary Figure 1). Interestingly, Kazakh and Mongolian cattle populations showed greater separation within these breeds by duplication. Unlike Qaidam, Xizang, and Yanbian cattle, Kazakh and Mongolian cattle may have less pressure of selection, which caused numerous meaningless duplications. These data suggest that artifical selection has shaped the CNVR diversity of each cattle breed during animal domestication.

### 3.3. Differentiated CNVRs between Qaidam cattle and other cattle breeds

We calculated the *V*_ST_ between Qaidam cattle (CDM) and other cattle breeds from the regions of North China (XZ, HSK, YB, and MG) (Supplementary Figure 2, Supplementary Tables 4–7). First, the top 1% signal value regions were kept out; then, it was annotated by the cattle reference genome (ARS-UCD1.2).

**Figure 3:**
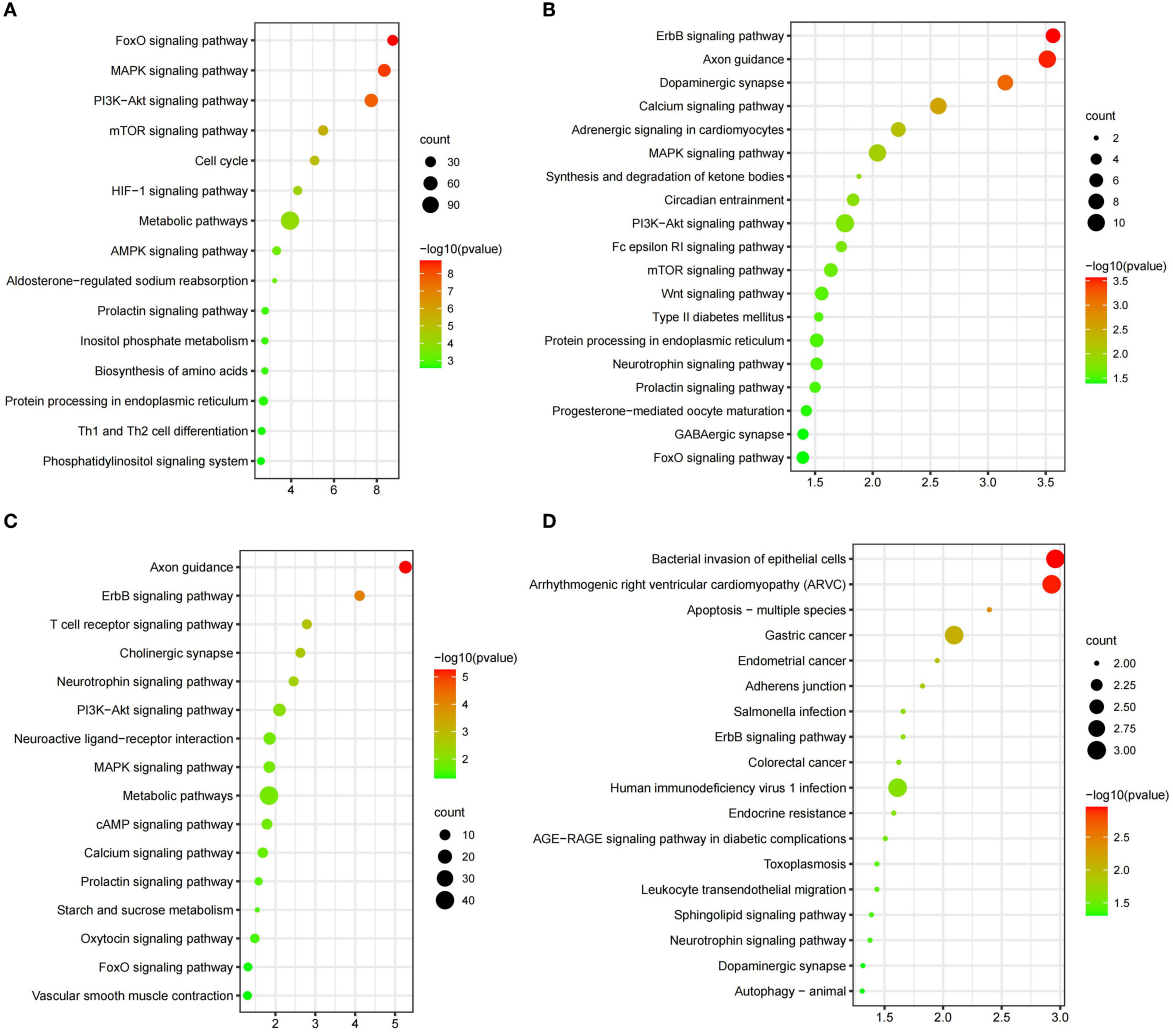
KEGG pathways from the enrichment analysis. **(A)** CDM vs. YB; **(B)** CDM vs. MG; **(C)** CDM vs. XZ; **(D)** CDM vs. HSK) (*p* < 0.05). We preferred showing part of the pathways; for more information, please see Supplementary Tables 8–11.

**Figure 4:**
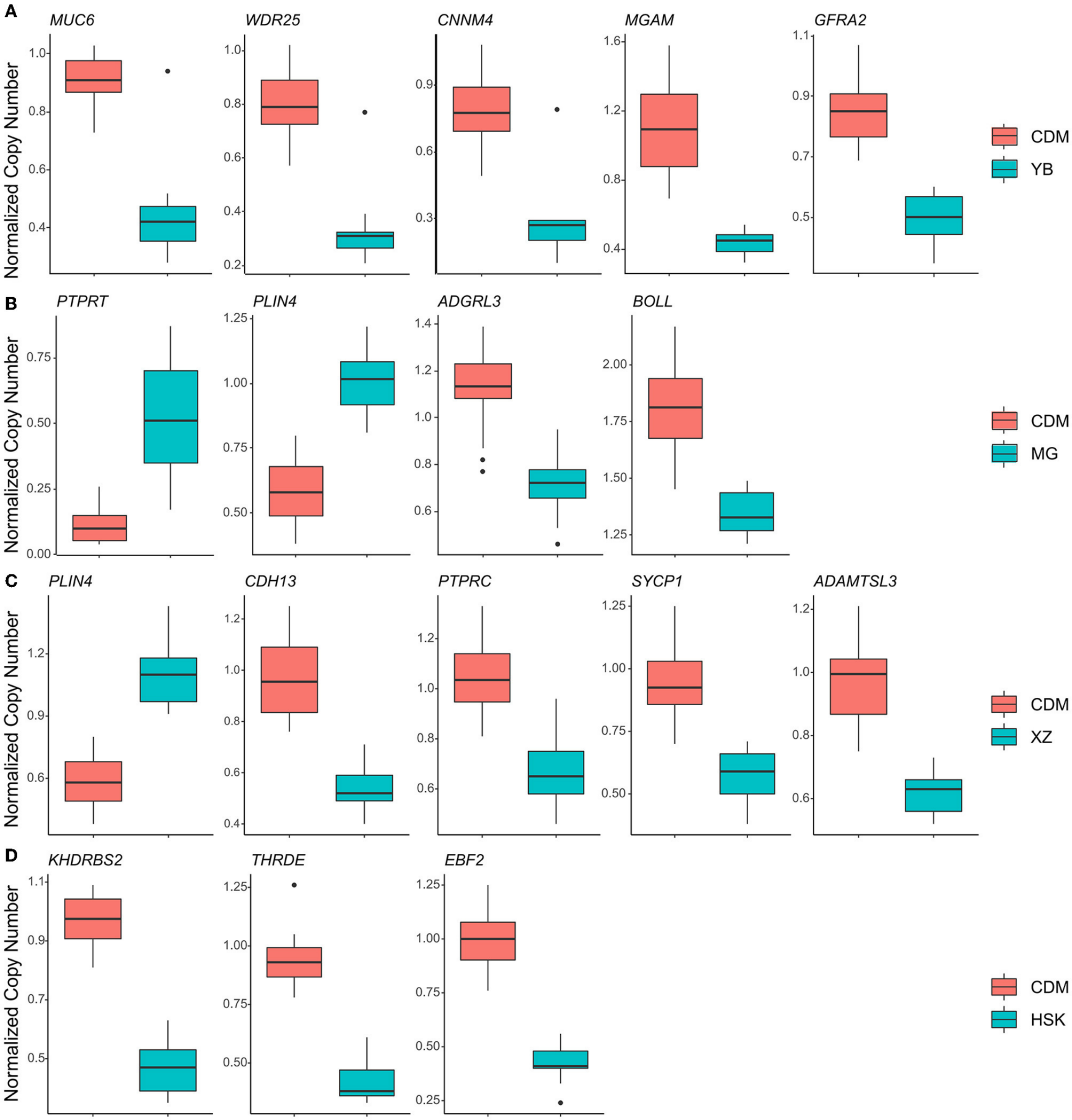
Normalized copy number comparison of the top 1% *V*_ST_ genes between CDM and other populations. **(A)** CDM compared with the YB population. **(B)** CDM compared with the MG population. **(C)** CDM compared with the XZ population. **(D)** CDM compared with the HSK population.

The selection signals between CDM and YB were enriched to “MAPK signaling pathway” (*p* = 4.49 × 10^−7^), “Pi3k-akt signaling pathway” (*p* = 1.82 × 10^−8^), “mTOR signaling pathway” (*p* = 3.22 × 10^−6^), “HIF-1 signaling pathway” (*p* = 4.95 × 10^−5^), and “aldosterone-regulated sodium reabsorption” (*p* = 5.91 × 10^−4^) (Figure 3A, Supplementary Table 8). There were five candidate genes (*MUC6, WDR25, CNNM4, MGAM*, and *GFRA2*) in the study (Figure 4A, Supplementary Table 4). The selection signals between CDM and MG were enriched to “ErbB signaling pathway” (*p* =2.73 × 10^−4^), “calcium signaling pathway” (*p* =2.69 × 10^−3^), “GnRH signaling pathway” (*p* = 0.01122), and “insulin signaling pathway” (*p* = 0.04588) (Figure 3B, Supplementary Table 9). Among these annotated genes, four genes (*PTPRT, BOLL, PLIN4*, and *ADGRL3*) deserved more attention in copy number between CDM and MG (Figure 4B, Supplementary Table 5). The selection signals between CDM and XZ were enriched to “axon guidance” (*p* = 5.48 × 10^−6^) and “ErbB signaling pathway” (*p* = 7.49 × 10^−5^) (Figure 3C, Supplementary Table 10). Among these annotated genes, five genes (*PLIN4, CDH13, SYCP1, PTPRC*, and *ADAMTSL3*) were noteworthy in copy number between CDM and XZ (Figure 4C, Supplementary Table 6). There was a difference in copy numbers between CDM and HSK. The selection signal enrichment pathways between CDM and HSK include “bacterial invasion of epithelial cells” (*p* = 1.10 × 10^−3^), “Salmonella infection” (*p* = 0.02195), and “human immunodeficiency virus 1 infection” (*p* = 0.02446) (Figure 3D, Supplementary Table 11). Among these annotated genes, three genes (*KHDRBS2, THRDE*, and *EBF2*) were notable in copy number between CDM and HSK (Figure 4D, Supplementary Table 7).

## 4. Discussion

During domestication and diversification, the frequency of copy number variation in the species' genome responds to selective pressure. Considerable effort has been applied to identify the causal mutations and genes. However, screening the selected genomic copy number genetic markers is complex. Over the past decades, high-throughput sequencing techniques and bioinformatics tools have been increasingly used to construct genome-wide CNV maps (1, 15, 17, 18). The diversity of CNVs has been extensively explored in *Bos Taurus, Bos Indicus*, and their crossing populations.

In our study, we investigated the CNV of 20 newly resequenced Qaidam cattle genomes based on the ARS_UCD 1.2 cattle reference genome. It improved the reliability of screening CNVs more than through UMD 3.1 assembly (19, 20). A total of 10,178 CNVRs were detected in five Chinese indigenous cattle breeds, and more than 99.9% CNVRs in length ranged from 1 to 100 kb. It was suggested that CNVs were widespread in Chinese cattle and may have been caused by the rapid adaptation during population expansion. For better statistics, variants were divided into three categories: duplication, deletion, and both duplication and deletion. The duplication was higher than deletions in number (Supplementary Table 3). And most of the CNVRs ranging from 2 to 5 kb in length (Figure 1A). In addition, the location of CNVRs is not uniformly distributed in the cattle genome (Figure 1C), and they are also not randomly distributed on chromosomes. The annotation uncovered that CNVRs are mostly annotated in the intergenic or intronic regions in the cattle genome. A previous study has also supported that many CNVRs are located on highly variable genes (15).

Compared to the analysis of the genome CNV in Qaidam cattle (18) for the first time, the role that CNVs have in the evolution of Qaidam cattle is becoming clear through our present study. The Qaidam cattle have strong adaptability to the arid environment, exhibiting dry, hypoxia, low air pressure, and large diurnal temperature difference (relative humidity 29–42%, precipitation 140–210.4 mm). Interestingly, YB cattle have almost opposite living conditions (relative humidity 68.6%; precipitation 500–700 mm) than Qaidam cattle. By consulting scientific articles, we found that *EBF1* and *ZNF521* related to fat development (21, 22) and *VEGFA, EGLN2*, and *ENO3* were associated with high altitude hypoxic adaptation (23–25). In the enrichment analysis, the *MGAM* gene was significantly enriched in the “metabolic pathways (bta01100, *P-*value = 0.000113)” (Figure 3A), and was also clustered in the “carbohydrate metabolic process (GO:0005975, *P-*value = 0.014482)” (Supplementary Table 8). A previous study reported on the CNVR overlapping with the *MAGM* gene, and that it was related to starch digestion (26). Specifically, we found that *MUC6* in Qaidam cattle was a normal-type CNVR, but it is a deletion CNVR in the YB cattle genome (Figure 4A). A previous study found CNV polymorphism in the *MUC6* gene of domestic sheep, and this CNVR presents normal or duplication under arid environments, and deletion in warm and humid environments (27). Structurally, large numbers of tandem repeats rich in Pro, Thr, and Ser residues in *MUC6* can affect the covalent attachment of O-glycans (28). In ruminants, such as sheep and cattle, the *MUC6* gene has been associated with gastrointestinal parasite resistance (29, 30). Therefore, we hypothesized that the copy number difference of the *MUC6* gene may influence the ability of antiparasitic immunity in Qaidam cattle and YB cattle.

High-quality beef is the breeding target of Qaidam cattle. In the comparison between Qaidam cattle and MG cattle (Supplementary Figure 2B), we observed that the *PRKCA, CAMK2D, PHKB*, and *GRID2* genes (Supplementary Table 5) (*V*_ST_ value > 0.43) were related to muscle growth and development by searching previous research studies (31–34). Moreover, we identified *PTPRT, BOLL, PLIN4*, and *ADGRL3* gene regions in the CNVRs of the top *V*_ST_ values which have obvious copy number differences between Qaidam cattle and MG cattle (Figure 4B). The *ADGRL3* gene is associated with the nervous system of the Fuzhong buffalo (34). In addition, *PTPRT* (Chr13: s17021.1) was associated with body weight for pre-weaning growth in Esme sheep (35). In addition, the functional enrichment analysis of candidate genes with top 1% signal *V*_ST_ values revealed that the “GnRH signaling pathway” and “calcium signaling pathway” were significantly overrepresented. These results imply that the selected genes might contribute to the characteristics of growth rate and meat quality in Qaidam cattle.

Body size is one of the important traits in the evaluation of beef selection. In this study, we identified *ADAMTSL3, PLIN4, CDH13, SYCP1*, and *PTPRC* genes of the top 1% signal regions between Qaidam and XZ cattle (Supplementary Table 6). According to previous research, *ADAMTSL3* plays an important role in chondrogenesis, morphogenesis, and skeletal growth in humans (36). A previous study reported that the bovine *ADAMTSL3* gene has specific polymorphisms in individuals and the SNPs (T1532C and C1899T) were significantly associated with body size traits (37). Our results further suggested that copy number in the *ADAMTSL3* gene may be one of the reasons for the difference in body size between XZ cattle and Qaidam cattle.

By comparing the copy number differences between the HSK and Qaidam breeds on the genome (Supplementary Figure 2C), we identified CNVRs with significant differences including *KHDRBS2, THRDE*, and *EBF2* (Figure 4D, Supplementary Table 7). One of the eye-catching genes was *EBF2*, which has copy number polymorphism and showed a normal type in Qaidam cattle but a deletion type in HSK cattle (Figure 4D). Previous studies showed that *EBF2* promotes brown adipocyte differentiation (38) and that its loss in mouse adipocytes abrogates brown adipose tissue (BAT) characteristics and function, leading to cold intolerance (39, 40). The cold tolerance of Qaidam cattle is an essential characteristic and it was speculated to be related to the copy number variation of *EBF2*.

## 5. Conclusion

Based on the high-quality *Bos taurus* reference genome, we constructed a CNV map of Northern Chinese Qaidam cattle using whole-genome resequencing data. Moreover, there are many copy number differences between Qaidam cattle and other cattle breeds from the regions of North China. It may play a crucial role in understanding the Qaidam cattle's adaptability, growth, and developmental characteristics In conclusion, these results provide a wealth of CNVR information to explore the valuable molecular markers in the Qaidam cattle genome.

## Data availability statement

The datasets presented in this study can be found in online repositories. The names of the repository/repositories and accession number(s) can be found in the article/Supplementary material.

## Ethics statement

The animal study was reviewed and approved by the Institutional Animal Care and Use Committee of Qinghai Academy of Animal Science and Veterinary Medicine, Qinghai University. Written informed consent was obtained from the owners for the participation of their animals in this study.

## Author contributions

YL drafted the manuscript and took part in the analysis of genome data. ZM, CL, and XW contributed to the sample collection. WW and YM performed the primary analysis of genome data. ZA and ZM revised the writing. ZM and CL designed the experiment and provided the funding for this research. All authors read and approved the final manuscript.
